# A Feasible and Efficacious Mobile-Phone Based Lifestyle Intervention for Filipino Americans with Type 2 Diabetes: Randomized Controlled Trial

**DOI:** 10.2196/diabetes.8156

**Published:** 2017-12-12

**Authors:** Melinda S Bender, Bruce A Cooper, Linda G Park, Sara Padash, Shoshana Arai

**Affiliations:** 1 Family Health Care Nursing Department School of Nursing University of California San Francisco San Francisco, CA United States; 2 Office of the Dean and Administration School of Nursing University of California San Francisco San Francisco, CA United States; 3 Community Health Services School of Nursing University of California San Francisco San Francisco, CA United States; 4 School of Nursing University of San Francisco San Francisco, CA United States

**Keywords:** randomized controlled trial, mobile health, Filipino American, type 2 diabetes, weight loss, physical activity, diet

## Abstract

**Background:**

Filipino Americans have a high prevalence of obesity, type 2 diabetes (T2D), and cardiovascular disease compared with other Asian American subgroups and non-Hispanic whites. Mobile health (mHealth) weight loss interventions can reduce chronic disease risks, but these are untested in Filipino Americans with T2D.

**Objective:**

The objective of this study was to assess feasibility and potential efficacy of a pilot, randomized controlled trial (RCT) of a culturally adapted mHealth weight loss lifestyle intervention (Pilipino Americans Go4Health [PilAm Go4Health]) for overweight Filipino Americans with T2D.

**Methods:**

This was a 2-arm pilot RCT of the 3-month PilAm Go4Health intervention (phase 1) with an active waitlist control and 3-month follow-up (phase 2). The waitlist control received the PilAm Go4Health in phase 2, whereas the intervention group transitioned to the 3-month follow-up. PilAm Go4Health incorporated a Fitbit accelerometer, mobile app with diary for health behavior tracking (steps, food/calories, and weight), and social media (Facebook) for virtual social support, including 7 in-person monthly meetings. Filipino American adults ≥18 years with T2D were recruited from Northern California. Feasibility was measured by rates of recruitment, engagement, and retention. Multilevel regression analyses assessed within and between group differences for the secondary outcome of percent weight change and other outcomes of weight (kg), body mass index (BMI), waist circumference, fasting plasma glucose, HbA1c, and steps.

**Results:**

A total of 45 Filipino American adults were enrolled and randomized. Mean age was 58 (SD 10) years, 62% (28/45) were women, and mean BMI was 30.1 (SD 4.6). Participant retention and study completion were 100%, with both the intervention and waitlist group achieving near-perfect attendance at all 7 intervention office visits. Groups receiving the PilAm Go4Health in phase 1 (intervention group) and phase 2 (waitlist group) had significantly greater weight loss, −2.6% (−3.9 to −1.4) and −3.3% (−1.8 to −4.8), respectively, compared with the nonintervention group, resulting in a moderate to small effect sizes (*d*=0.53 and 0.37, respectively). In phase 1, 18% (4/22) of the intervention group achieved a 5% weight loss, whereas 82% (18/22) maintained or lost 2% to 5% of their weight and continued to maintain this weight loss in the 3-month follow-up. Other health outcomes, including waist circumference, BMI, and step counts, improved when each arm received the PilAm Go4Health, but the fasting glucose and HbA1c outcomes were mixed.

**Conclusions:**

The PilAm Go4Health was feasible and demonstrated potential efficacy in reducing diabetes risks in overweight Filipino Americans with T2D. This study supports the use of mHealth and other promising intervention strategies to reduce obesity and diabetes risks in Filipino Americans. Further testing in a full-scale RCT is warranted. These findings may support intervention translation to reduce diabetes risks in other at-risk diverse populations.

**Trial Registration:**

Clinicaltrials.gov NCT02290184; https://clinicaltrials.gov/ct2/show/NCT02290184 (Archived by WebCite at http://www.webcitation.org/6vDfrvIPp)

## Introduction

### Background

As the fastest growing US racial/ethnic group, Asian Americans represent 6.4% (approximately 21 million) of the US population [1] and are at a high risk for early development of type 2 diabetes (T2D) at lower body mass index (BMI) than non-Hispanic whites [2]. Filipino Americans (FA) are the third largest US Asian subgroup (2,717,844) and the largest California Asian population (1,474,707) [3]. Filipino Americans have the highest burden and prevalence of obesity and T2D among Asian American subgroups and non-Hispanic whites, and have early cardiovascular-metabolic disease risk, with higher mortality rates [4]. Yet, there is limited and incipient preventive health research focused on the Filipino American health disparity [5,6]. Thus, it is imperative to identify effective interventions to reduce these critical health disparities.

Weight loss lifestyle interventions promoting increased physical activity (PA) and a healthy diet (with as little as 5%-7% weight loss) have been shown to reduce obesity and related T2D risks by 58% [7]. The American Heart Association and Healthy People 2020 recommends such interventions, particularly for high-risk racial/ethnic minority populations [8-10]. However, intensive lifestyle interventions, such as the Diabetes Prevention Program (DPP) requiring 16 sessions, may be burdensome for participants and labor intensive to deliver [7]. Alternatively, education, coaching, and social support can be delivered virtually via the Internet, providing real-time feedback promoting adherence to healthy behaviors. Combining mobile health (mHealth) technologies, including commercially available apps and PA trackers (eg, pedometers), offers optional intervention delivery mechanisms that can be scalable and cost-effective [11].

US demographics support the delivery of lifestyle intervention programs via mHealth technology. Approximately 95% of US adults own a mobile phone (77% smartphones) and 76% access Facebook daily [12]. A recent study found that Filipino AmericansA (81.7%) ranked highest for mobile technology ownership and usage compared with whites (69.9%) [13]. A systematic review found mHealth interventions to be beneficial for increasing PA and weight loss [14] and effective for T2D self-management [15]. A meta-analysis found that mHealth weight loss interventions had a medium effect size of 0.43, supporting its continued development and use with lifestyle interventions [16].

### Objective

Therefore, we conducted a pilot randomized controlled trial (RCT) called the Pilipino (ie, Filipino) Americans Go4Health (PilAm Go4Health). PilAm Go4Health was an mHealth culturally adapted weight loss lifestyle intervention promoting PA and healthy eating for Filipino Americans with obesity and T2D to reduce subsequent cardiovascular risks. The purpose of this paper is to report the feasibility of PilAm Go4Health (measured by recruitment, engagement, and retention) and potential efficacy (measured by percent weight and weight [kg] change). Positive findings will support a follow-on full-scale RCT to test the effectiveness of a culturally adapted mHealth weight loss lifestyle intervention for Filipino Americans with T2D. Qualitative assessments from participants’ responses of the PilAm Go4Health’s acceptability and cultural relevance (measured by process evaluations and postprogram interviews) were previously reported [17].

## Methods

### Design

This was a pilot RCT of the PilAm Go4Health, a 3-month culturally adapted mHealth weight loss lifestyle intervention for Filipino Americans with obesity and T2D, followed by a 3-month follow-up maintenance period. This 2-arm trial consisted of an intervention group and an active waitlist control (waitlist) group. Institutional approval from the Committee on Human Research was obtained before the implementation of the study. Before enrollment, all participants provided written informed consent.

PilAm Go4Health consisted of a weight loss lifestyle intervention based on the DPP [7] that was modified to incorporate mobile technologies (Fitbit accelerometer plus app with diary) and private Facebook group for healthy behaviors tracking, real-time feedback, coaching, and virtual social support. The overall PilAm Go4Health weight loss goal was a 5% weight reduction from baseline by 3 months.

### Participants

Participants were recruited from the San Francisco Bay Area from December 2014 to December 2015. Recruitment was primarily through word of mouth, community events, and snowball methods.

**Figure 1:**
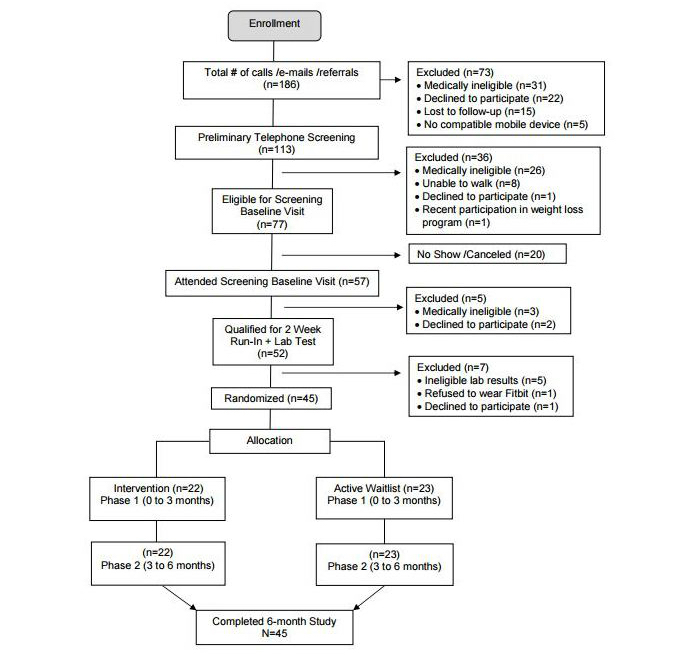
Consolidated Standards of Reporting Trials (Consort) flow diagram.

Online recruitment strategies included the following: San Francisco Bay Area Craigslist (a San Francisco company providing websites for local classified ads of sale items and services), a dedicated study Facebook website, and an institutional website. Complete recruitment details are published elsewhere [18]. Those who met the screening and eligibility criteria (N=45) were enrolled and randomized into the study (Figure 1).

#### Inclusion Criteria

Eligibility was based on the DPP criteria and American Heart Association metabolic syndrome risks [7,19]. Key inclusion criteria were self-identified Filipino; ≥18 years; BMI >23 kg/m^2^ for Asians; physician diagnosis of T2D (non-insulin dependent); own a smartphone, tablet, or laptop with Internet access; and English language proficient.

#### Exclusion Criteria

Exclusions included disabilities precluding walking for 20 min; on a special exercise program; participation in a weight loss program in the past year; uncontrolled T2D (fasting plasma glucose >200 mg/dL); endocrine or glucose metabolism associated disease (eg, Cushing syndrome or polycystic ovary syndrome); and uncontrolled hypertension. A detailed list of screening and eligibility criteria are reported in a previous publication [18].

### Theoretical Framework

Social cognitive theory and the transtheoretical model for health behavior change helped to guide the study design [20,21]. According to the social cognitive theory, role models along with sociocultural and environmental feedback (positive or negative) can influence engagement and adherence to healthy lifestyle behaviors, including healthy eating and PA. Social support may also enhance self-efficacy for healthy weight loss behaviors. To enhance social support, PilAm Go4Health incorporated a private Facebook group and welcomed family members to in-person research office visits.

The transtheoretical model posits that health behavior change involves progress through 6 stages of change: precontemplation, contemplation, preparation, action, maintenance, and termination [21]. Applied research has demonstrated dramatic improvements in recruitment, retention, and engagement using stage-matched interventions and proactive recruitment procedures. To confirm that potentially eligible participants were stage-matched (ie, preparation for change stage) with the PilAm Go4Health, we incorporated a 2-week run-in period to assess readiness for change to help facilitate assessment of both feasibility and potential efficacy of this intervention program.

### Cultural Adaptation

Before the study, the PilAm Go4Health was culturally tailored for Filipino Americans according to recommended published cultural adaptation guidelines [22] that include the following 5 components: (1) peripheral, (2) evidential, (3) constituent involving, (4) sociocultural, and (5) linguistics. Examples of each are provided in Table 1. A comprehensive description of the adaptation strategies used in the study is provided in a previous publication [18].

### Screening Baseline Visit and Run-In Period

Eligible participants who passed the telephone screening were invited for a screening baseline visit that included a physical exam (weight, height, BMI, waist and hip circumference, and blood pressure), fasting blood draw (eg, fasting plasma glucose and hemoglobin A1c), and questionnaires. Those who passed the screening baseline visit and fasting blood draw received a Fitbit Zip accelerometer and Fitbit app with diary with training and were then enrolled in a 14-day run-in period.

A study run-in period was incorporated to assess whether participants were in the transtheoretical model’s *readiness or preparation for change stage* [21]. The run-in period was designed to screen out potential noncompliant participants. Although this may minimize the sample size, it increases the statistical power [24] to reduce the possibility of erroneously rejecting the PilAm Go4Health as a potentially efficacious weight loss intervention. This helps to determine whether the intervention was feasible (acceptable and practical) and potentially efficacious (able to generate beneficial results under ideal circumstances) [25].

Participants in the run-in period were asked to wear the Fitbit Zip daily for at least 10 hours/day and send photos of all food and drinks consumed for 3 consecutive days. Those who complied at least 70% of the time with the run-in requirements demonstrated readiness for behaviors change and were enrolled and randomized into the study. Further details on the run-in protocol were previously published [18].

### Randomization

A total of 45 participants were enrolled and randomized in a 1:1 ratio (computer-generated random allocation sequence) and then stratified by gender in permuted randomly selected block sizes of 2 and 4 to an intervention group (n=22) or an active waitlist group (n=23; see Figure 1). Due to the nature of a lifestyle intervention, only the lab technicians and statistician were blinded, but research investigators, staff, and participants were not.

### Intervention Group

Trained research staff implemented the PilAm Go4Health intervention. In phase 1 (baseline to 3 months) immediately after randomization, intervention participants were trained on using the Fitbit accelerometer to self-monitor real-time PA steps and associated app with diary to self-report daily food/calorie intake and weekly weight. They joined the study’s private Facebook group for virtual social support, coaching, and weekly education topics posted by research staff. Participants were encouraged to join the Facebook discussions at least once a week. At this training visit, they were given tailored short- and long-term weight loss goals based on the participant’s baseline weight, PA, and diet information. Depending on their progress with tailored goals, research staff provided each participant tailored feedback, coaching, and support during research office visits at 1, 2, and 3 months. Table 2 outlines the PilAm Go4Health components delivered at each visit and weekly Facebook discussion topics posted by research staff.

In phase 2 at the 3-month office visit, intervention participants transitioned to a 3-month follow-up and were removed from the private Facebook group. Participants were asked to continue using their Fitbit and app with diary to track health behaviors and maintain their weight loss goals. Follow-up office visits were scheduled at 4 and 6 months. Intervention participants completed the study at 6 months. Further PilAm Go4Health intervention details are reported elsewhere [18].

**Table 1:** Examples of Pilipino Americans Go4Health [PilAm Go4Health] cultural adaptation strategies.

Components	Example
Peripheral	Photos of common Filipino foods were used in Filipino food pamphlet
Evidential	Health education sessions included information on the high prevalence of and factors associated with type 2 diabetes among Filipino Americans
Constituent involving	Filipino American community stakeholder (leaders, members, organizations, and health providers) input from individual interviews and focus group helped to inform the study design
Sociocultural	To align with a Filipino American family-centric culture, family members were welcome to attend the participant’s scheduled office visits
Linguistics	Healthy lifestyle education pamphlets translated in Tagalog for Filipino Americans were provided by the National Heart, Lung, and Blood Institute [23]

**Table 2:** Pilipino Americans Go4Health [PilAm Go4Health] intervention sessions (physical exam includes height, weight, body mass index, waist circumference, and blood pressure).

Schedule		Lifestyle education and coaching
**Phase 1 (baseline to 3 months) Pilipino Americans Go4Health [PilAm Go4Health] intervention**		
	Baseline visit (individual)	Lifestyle balance and social networking
		Initiating physical activity and healthy eating plan with short- and long-term goals for weight loss
		Physical exam, blood draw, and surveys
		Fitbit Zip, app and diary training for tracking steps, food/calories, and weight and private Facebook group training
	1-month visit (family members welcome)	Progress report and coaching on healthy behaviors
		Benefits and ways to be physically active
		Social support for physical activity
		Filipino dancing (Zumba, cha cha), basketball, and walking
		Monitoring physical activity steps
	2-month visit (family members welcome)	Progress report and coaching on healthy behaviors
		Benefits and ways of healthy eating and limiting fat
		Social support for healthy eating
		Healthy Filipino food alternatives and recipes
		Monitoring weight
	3-month visit (family members welcome)	Progress report + relapse prevention, problem-solving, and staying motivated
		Handling barriers to healthy behaviors
	Transition to phase 2	Social support for maintaining healthy behaviors
		Physical exam, blood draw, and surveys
		Postintervention process evaluation interview
**Private Facebook group**		
	Baseline to 3 months only; removed from Facebook group at 3-month visit	Research staff monitored and posted 12 weekly discussions covering topics such as the following: benefits of regular exercise, healthy fruits and vegetables, water and low-calorie drinks, tracking weight, healthy recipes, handling barriers to weight loss and healthy lifestyle behaviors, benefits of social support for weight loss, maintaining glycemic control, and medication adherence
		Weekly prompts to post and share photos, recipes tried, progress reports or barriers encountered, and encouragement for Facebook peers
**Phase 2 (3 to 6 months) follow-up maintenance**		
	4-month visit (family members welcome)	Progress report, continue using Fitbit and app with diary to track steps, food, and weight
		Personal Facebook support group
		Reviewed relapse prevention, maintaining healthy behaviors, and dealing with barriers to healthy lifestyle
	6-month visit (individual)	Progress report and relapse prevention, handling barriers
		Coaching to maintain healthy behaviors
		Physical exam, blood draw, and surveys
		Poststudy process evaluation interview

### Waitlist Control Group

In phase 1 at the baseline randomization visit, waitlist participants received only the Fitbit accelerometer and training for daily wear. They returned for 1- and 3-month office visits when they received hepatitis B and C education, respectively.

In phase 2 at the 3-month office visit, waitlist participants transitioned to receive the PilAm Go4Health intervention and returned for 3 office visits at 4, 5, and 6 months (Table 2). Waitlist participants completed the study at 6 months.

### Data Collection

All participants’ anthropometric measures—weight (kg), height, BMI (kg/m^2^), waist circumference (cm), blood pressure, serum labs (eg, plasma glucose and HbA1c)—were collected at baseline, 3 months, and 6 months during research office visits and stored in secure study data servers. All Fitbit steps and self-reported app and diary data (calorie/food and weight) were wirelessly uploaded and transmitted in real time directly to secure Fitbit data servers. Each participant’s secure Fitbit account was also linked to a secondary Fitabase account (Fitabase, a San Diego based corporation) where data were uploaded and stored on secure confidential Fitabase data servers [26]. Facebook group data were wirelessly uploaded to secure Facebook data servers. All participant data were subsequently uploaded to secure institutional study data servers. Only approved research staff and investigators had access to study data. For further details on data collection protocol, see previous publication [18].

### Outcome Measures

#### Primary Outcome

Feasibility was based upon three criteria: recruitment, engagement, and retention. Recruitment goal was to have 45 eligible participants recruited, enrolled, and randomized for this study. Engagement goal was to have participants attend 5 out of 7 intervention office visits (receipt of 9 of the 16 DPP sessions) as a measure for completing the program. This threshold was based on the Centers for Disease Control and Prevention’s (CDC) required number of DPP sessions considered for program completion [27]. Retention goal was to have at least 80% of randomized participants complete the study, as defined by attending 5 of 7 office visits and complete all required study assessments (physical exams, labs, and surveys) at baseline, 3-month, and 6-month visits.

We monitored adherence to tracking target health behaviors using the Fitbit Zip and Fitbit app with diary. These additional engagement measures described the uptake and acceptance of the PilAm Go4Health program by participants. The criteria for mobile technology tracking by participants were as follows: (1) logging weight at least once/week, (2) logging daily food/calories at least once/week, and (3) wearing Fitbit Zip at least 5 days/week. However, currently there are no standard thresholds for frequency of mHealth app use to evaluate feasibility of an intervention. Any such thresholds would be arbitrary. Therefore, we chose not to use adherence as a measure of engagement to evaluate feasibility.

#### Secondary Outcome

Percent weight change was used to assess potential efficacy during phase 1 and 2 for each arm. In phase 1, the intervention group received the PilAm Go4Health from baseline to 3 months, whereas the waitlist group only used the Fitbit Zip without coaching. In phase 2, the waitlist group received the PilAm Go4Health from 3 to 6 months, whereas the intervention group transitioned to the follow-up maintenance phase.

#### Other Outcomes

For each arm, change in weight (kg) was measured weekly for 6 months, and change in BMI, waist circumference, fasting plasma glucose, and HbA1c were measured at baseline, 3 months, and 6 months, whereas daily step counts were measured in real time via the Fitbit Zip.

### Statistical Analysis

Descriptive analyses for demographic, clinical, and outcome measures were computed using IBM SPSS for Windows version 24. Descriptive statistics were obtained by using *t* test, Mann-Whitney *U* test, Wilcoxon signed-rank test, or chi-square test for continuous, nonparametric, or categorical variables as appropriate. Between-group differences in percent weight change categories over time were analyzed using a bootstrap chi-square test, including the Mantel-Haenszel test of trend.

The feasibility outcome for recruitment was based upon achieving the target sample size. We reported the simple proportion (%) of participants within each randomized group who met the various target behavior threshold criteria for engagement and retention during the 3-month PilAm Go4Health.

The question for each secondary outcome was whether the change during both study phases was greater for the group receiving the PilAm Go4Health than the nonintervention group. Multilevel regression (aka linear mixed models or hierarchical linear models) was employed to test differences between the 2 arms in their change trajectories. This effect is also called the cross-level interaction between time and group [28,29]. In addition to the primary test of between-group change, the simple slopes were also tested to determine whether the change was significant *within* each group.

For these analyses, there were no missing data for the 2 groups. Therefore, a multilevel regression models approach was used over more traditional repeated measures analysis of variance (since missing data were not an issue) [28,29]. The use of multilevel regression allowed for the use of bootstrapping when the assumption of normality was not tenable. Bootstrapped full information maximum likelihood models were estimated to obtain nonparametric, bias-corrected bootstrapped CIs (BC CI) for estimation and inference regarding hypotheses [30-32]. These analyses were carried out with Stata/SE version 14 [33,34]. Primary analysis included intention to treat. Significance was evaluated using a 2-sided alpha of .05.

## Results

### Sociodemographic Results

A total of 113 potential participants were screened; 45 were eligible, enrolled, and randomized (see Figure 1). Mean age was 57.6 (SD 9.8), with 62% (28/45) female. The majority were immigrants (38/45; 84%). Overall, participants were categorized as obese with mean BMI 30.1 (SD 4.6) (Table 3). The only sociodemographic variable with a difference between the 2 groups was “Years lived in the United States” (often used as a proxy for acculturation). Although a majority of participants were immigrants, they were highly acculturated (Marin Acculturation Scale [35], mean score=3.5). As there were no between-group differences in acculturation scores, the outcome analyses were not adjusted for years lived in the United States.

### Primary Outcomes

Results of all primary outcomes indicated that the PilAm Go4Health intervention program was feasible. For the study, 45 eligible participants were recruited, passed the run-in period, and enrolled and randomized over a 1-year period (Figure 1). Word of mouth was the dominate recruitment strategy that yielded the highest number of potential participants, followed by in-person invitation to join the study at local Lion’s Club faith-based weekly health fairs. Feasibility engagement was measured by attendance at intervention office visits. Both the intervention and waitlist group achieved near-perfect attendance at all 7 intervention office visits (95% [21/22] and 100% [23/23], respectively), well above the standard CDC threshold for DPP completion. Finally, all 45 participants (100%) completed the study at 6 months, meeting the retention rate goal (Table 4).

Adherence to additional mHealth engagement measures, including logging weight and food/calories and wearing the Fitbit, was similar between the intervention and waitlist groups. With the exception of logging weight at least once/week, both groups demonstrated relatively high adherence to tracking weekly health behaviors in excess of 80% of the time when they received the 3-month intervention (Table 4).

**Table 3:** Pilipino Americans Go4Health [PilAm Go4Health] participant baseline sociodemographics, anthropometrics, and serum labs.

Variable		Overall (N=45)	Intervention (n=22)	Waitlist (n=23)	*P* value
Age in years, mean (SD^a^)		57.6 (9.8)	57.4 (9.8)	57.7 (10.0)	.90
Race (Filipino), n (%)		45 (100)	22 (100)	23 (100)	.99
Gender (female), n (%)		28 (62)	14 (63)	14 (60)	.85
**Marital status, n (%)**					.06
	Never married	5 (11)	1 (5)	4 (17)	
	Divorced/widowed	10 (22)	7 (32)	3 (13)	
	Married/cohabitating	30 (67)	14 (64)	17 (70)	
**Education, n (%)**					.67
	College 1-4 years	36 (80)	18 (82)	18 (78)	
	Graduate school	9 (20)	4 (18)	5 (22)	
**Employed, n (%)**					.21
	Full or part time	31 (69)	17 (77)	14 (61)	
	Unemployed	2 (4)	1 (5)	1 (4)	
	Retired, n (%)	12 (27)	4 (8)	8 (35)	
**Years lived in the United States, n (%)**					.003
	US born	7 (16)	0 (0)	7 (30)	
	≥5-10+ years	38 (84)	22 (100)	16 (70)	
**Marin acculturation score**					
	Mean (SD)	3.5 (0.6)	3.5 (0.6)	3.5 (0.7)	.91
	Low score <2.99, n (%)	9 (20)	4 (18)	5 (22)	.77
	High score >2.99, n (%)	36 (80)	18 (82)	18 (78)	
**Weight in kg**					
	Mean (SD)	75.8 (15.4)	72.6 (10.8)	78.8 (18.6)	.19
	Median	74.5	72.7	74.9	
Body mass index in kg/m^2^ (SD)		30.1 (4.6)	28.6 (3.6)	31.5 (5.1)	.03
Waist circumference in cm (SD)		99.6 (10.7)	97.1 (8.7)	101.9 (12.1)	.13
Fasting glucose in mg/dL (SD)		135.3 (25.8)	133.0 (20.8)	137.4 (30.1)	.57
HbA1c, % (SD)		7.42 (0.87)	7.39 (0.82)	7.44 (0.93)	.84
Steps per day (SD)		7101 (2391)	7483 (2416)	6736 (2363)	.30

^a^SD: standard deviation.

**Table 4:** Pilipino Americans Go4Health [PilAm Go4Health] office visit attendance and adherence to target health behaviors by group.

Target behaviors (N=45)	Intervention group (n=22) rate of adherence (0 to 3 months) n (%)	Waitlist control group (n=23) rate of adherence (3 to 6 months) n (%)
Attended all 7 intervention office visits	21 (95)	23 (100)
Logging weight at least once/week^a^	17 (79)	15 (64)
Logging food/calorie intake at least once/week^a^	20 (89)	19 (83)
Wear the Fitbit at least 5 days/week^a^	21 (97)	21 (91)

^a^Adherence signifies weekly mean of participants adhering to target behavior over the 12-week intervention period.

### Secondary Outcomes

The results of the analysis for the main secondary outcome (percent weight change) are compelling, as are the results of the other secondary outcomes (Table 5). *All statistically significant (indicated by no zero in 95% BC CI) simple slopes and cross-level interactions are highlighted in italicized type*. The estimated simple slopes in Table 5 represent *within*-group changes, and cross-level interactions represent the *between*-group differences.

**Figure 2:**
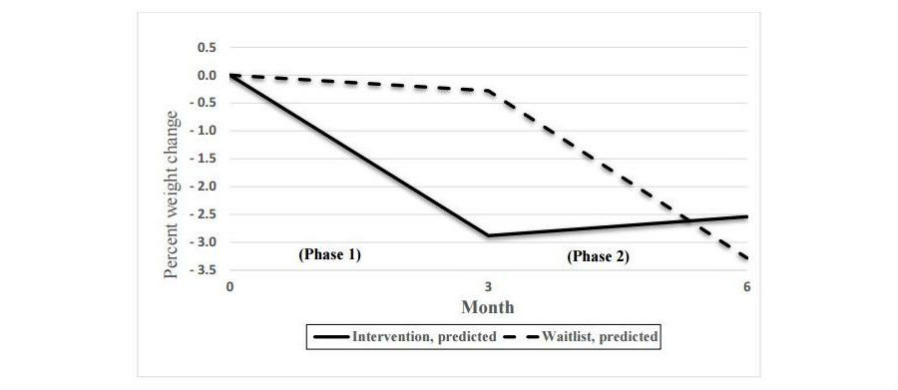
Percent weight change over 6 months by group—multilevel regression (phase 1—intervention group received PilAm Go4Health [Pilipino Americans Go4Health] weight loss intervention; phase 2—waitlist control group received PilAm Go4Health weight loss intervention).

All the cross-level interactions for phase 1 were significant and in the expected direction (Table 5). For example, in the column for the cross-level interaction, the point estimate for weight shows that the decrease was 2 kg greater for the intervention group than for the waitlist group. The BC CI for the 2 kg decrease shows that the population difference might be as great as 3 kg or as small as 1.1 kg, but it is not 0. This weight change had a moderate effect size of 0.53 (Cohen *d*). Close examination of the simple slope for the intervention group was significant but not that of the waitlist group—just what we would expect. The intervention group’s weight loss was equivalent to a significant 2.9% loss in their baseline weight (BC CI: −3.9 to −2.0). This is in contrast in to the waitlist group’s insignificant 0.3% loss in their baseline weight (Figure 2).

As one might expect in phase 2, when the intervention group transitioned to the follow-up and the waitlist group received the PilAm Go4Health intervention program, the results were inverted. Waitlist group’s mean weight decreased 2.5 kg more than the intervention group (BC CI: 1.4 to 3.5). This is between a weak and medium effect (Cohen *d*=0.37). The intervention group’s simple slope showed a trivial 0.28 kg increase in weight (BC CI: −.24 to .83), whereas waitlist group’s phase 2 simple slope decreased 2.2 kg (BC CI: −3.1 to −1.3). In phase 2, the cross-level interaction showed a 3.3% greater decrease for the waitlist group (BC CI: −1.8 to −4.8), and the simple slope for the waitlist group’s 3.0% decrease in weight loss was significant (BC CI: −4.2 to −1.7), but the intervention group’s increase of 0.35% in the simple slope was not (BC CI: −.37 to 1.1).

#### Intervention Group—Percent Weight Loss Goals Achieved

The overall PilAm Go4Health weight loss goal was a 5% weight reduction. In phase 1, about 18% (4/22) of the intervention group achieved a 5% weight loss, whereas 82% (18/22) of the group’s remaining participants maintained or lost 2% to 5% of their weight. During maintenance in phase 2, over 90% (20/22) of the intervention group continued to maintain or lose 2% to 5% more weight (Table 6).

#### Waitlist Group—Percent Weight Loss Goals Achieved

In phase 1, over 83% (19/23) of the waitlist group maintained or gained 2% to 5% more weight (see Table 6). This pattern was reversed in phase 2 with 70% (16/23) of the waitlist participants receiving PilAm Go4Health having maintained or lost between 2% and 5% of their weight. Most notably, 30% (7/23) of waitlist participants achieved the 5% weight loss goal, almost twice that of the phase 1 intervention group.

#### Other Outcomes

A similar pattern of weight effects was also observed for other outcomes, waist circumference, BMI, and step counts, with mixed improvements in fasting glucose and HbA1c (Table 5). Significant cross-level interactions were detected for fasting glucose in both phases for the PilAm Go4Health activated groups. The simple slope (within group) for the intervention group was *significant*, indicating that the fasting glucose value had *increased* significantly during follow-up (10.7 mg/dL [3.4-18.5]). However, the simple slope for the waitlist group was not significant in phase 2, although it was in the expected direction (−8.9 mg/dL [−21.0 to 1.7]).

Opposing and mixed patterns were displayed in the HbA1c’s outcomes. In phase 1, the intervention group’s cross-level interaction was not significant, although the group’s simple slope was significant and in the expected direction (−.49% [BC CI: −.80 to −.21]). In contrast, the waitlist group’s HbA1c’s cross-level interaction in phase 2 was significant and in the expected direction, but not the simple slope, although it was in the expected direction.

Overall step counts significantly increased for each study arm that received the PilAm Go4Health in phase 1 and phase 2 compared with the nonintervention group. The greater number of assessments (14 weeks in phase 1; 13 weeks in phase 2) allowed for a more sensitive examination of the linear and quadratic components of change for the 2 groups and phase-related changes in trajectories. The cross-level interactions and simple slopes for both linear and quadratic slopes were significant for the PilAm Go4Health intervention group for phase 1 and the PilAm Go4Health waitlist group during phase 2 with expected significant large effect sizes.

**Table 5:** Pilipino Americans Go4Health [PilAm Go4Health] multilevel regression analyses of secondary outcomes for phase 1 (baseline to 3 months) and phase 2 (4 to 6 months) (N=45; intervention group: n=22; and waitlist group: n=23). All statistically significant (indicated by no zero in 95% BC CI) simple slopes and cross-level interactions are highlighted in italicized type.

Outcome measures		Intervention^a^ mean (SD^b^)	Intervention simple slopes^c^ (95% BC CI)^d^	Waitlist^a^ mean (SD)	Waitlist simple slopes^c^ (95% BC CI)^d^	Cross-level interactions^e^ (95% BC CI)^d^	Effect size Cohen *d*
**Percent weight change**							
	P1 (phase 1)	−2.9 (2.4)	− *2.9 (−3.9 to −2.0)*^f^	−0.28 (2.0)	−.28 (−1.0 to .56)	− *2.6 (−3.9 to −1.4)*	
	P2 (phase 2)	−2.5 (3.0)	.35 (−.37 to 1.1)	−3.3 (3.4)	− *3.0 (−4.2 to −1.7)*^f^	− *3.3 (−1.8 to −4.8)*	
**Weight (kg)**							
	BL (Baseline)	72.6 (10.8)		78.8 (18.6)			
	P1	70.5 (10.6)	− *2.1 (−2.9 to −1.4)*^f^	78.6 (19.2)	−.12 (−.72 to .59)	− *2.0 (−3.0 to −1.1)*	0.53
	P2	70.8 (11.0)	.28 (−.24 to .83)	76.4 (19.8)	− *2.2 (−3.1 to −1.3)*^f^	*2.5 (1.4 to 3.5)*	0.37
**Body mass index (kg/m^2^)**							
	BL	28.5 (3.6)		31.5 (5.1)			
	P1	27.7 (3.6)	− *.81 (−1.1 to −.56)*^f^	31.5 (5.5)	−.05 (−.29 to .24)	− *.77 (−1.2 to −.41)*	
	P2	27.8 (3.6)	.10 (−.11 to .31)	30.5 (5.6)	− *.92 (−1.3 to −.51)*^f^	*1.0 (.55 to 1.5)*	
**Waist circumference (cm)**							
	BL	97.1 (8.7)		101.9 (12.1)			
	P1	94.6 (9.2)	− *2.5 (−3.8 to −1.4)*^f^	102.1 (12.4)	.16 (−1.1 to 1.5)	− *2.7 (−4.5 to −.91)*	
	P2	94.2 (9.5)	−.43 (−1.5 to .54)	99.9 (13.0)	− *2.2 (−3.5 to −1.1)*^f^	*1.8 (.23 to 3.4)*	
**Fasting glucose (mg/dL)**							
	BL	133 (20.8)		137.4 (30.1)			
	P1	118 (20.3)	− *15 (−25 to −5.3)*^f^	141.0 (32.1)	3.5 (−4.2 to 11.2)	− *18.5 (−31.4 to −6.5)*	
	P2	128.7 (30.6)	*10.7 (3.4 to 18.5)*	132.0 (33.0)	−8.9 (−21.0 to 1.7)^f^	*19.6 (6.7 to 33.6)*	
**HbA1c (%)**							
	BL	7.4 (0.82)		7.4 (0.93)			
	P1	6.9 (0.67)	− *.49 (−.80 to −.21)*^f^	7.3 (1.0)	−.14 (−.41 to .05)	−.34 (−.70 to .04)	
	P2	7.1 (0.98)	.15 (−.03 to .37)	7.1 (1.2)	−.18 (−.42 to .07)^f^	.32 (.01 to .64)	
**Step counts**							
	P1 Linear	7483 (2415)	*L560 (210 to 862)* ^f^	6735 (2363)	L −93 (−205 to 15)	*L654 (275-975)*	1.74
	Quadratic	10,178 (4593)	Q −35 (−56 to 13)^f^	6469 (2936)	Q 2.4 (−6.6 to 11.8)	*Q −37 (−60 to −14)*	
	P2 Linear	9524 (3626)	L −206 (−477 to 39)	7208 (2719)	*L 403 (56-770)* ^f^	*L −610 (−1064 to −187)*	1.44
	Quadratic	8546 (4416)	Q10.9 (−6.2 to 29.3)	7538 (4025)	*Q −27 (−55 to −2)* ^f^	*Q 38 (7.8 to 72)*	

^a^Observed values.

^b^SD: standard deviation.

^c^Estimated simple slope.

^d^Nonparametric bias-corrected bootstrapped CI (BC CI) is significant if “0” not in confidence interval.

^e^Difference between groups.

^f^Received PilAm Go4Health.

**Table 6:** Percentage weight change achieved by group (N=45).

Phases		Gained ≥2% to <5% n (%)	Stable + <2% n (%)	Lost ≥2% to <5% n (%)	Lost ≥5% to <10% n (%)	*P* value^a^
**Phase 1**						.001
	Intervention^b^ (n=22)	0 (0)	10 (45)	8 (36)	4 (18)	
	Waitlist (n=23)	4 (17)	15 (65)	4 (17)	0 (0)	
**Phase 2**						.001
	Intervention (n=22)	2 (9.1)	16 (72)	4 (18)	0 (0)	
	Waitlist^b^ (n=23)	0 (0)	10 (43)	6 (26)	7 (30)	

^a^*P* value for Mantel-Haenszel chi-square test for trend.

^b^Received PilAm Go4Health.

## Discussion

### Key Findings

The PilAm Go4Health was feasible as measured by achieving the recruitment, engagement, and retention threshold goals. Results demonstrated potential efficacy of the PilAm Go4Health in reducing weight in Filipino Americans with overweight and T2D. Each group receiving the PilAm Go4Health program (intervention group in phase 1 and waitlist group in phase 2) demonstrated significant weight loss, underscoring the PilAm Go4Health potential efficacy. In phase 1, over half of the intervention participants lost weight. Although only 18% (4/22) achieved the overall 5% weight loss goal by 3 months, the weight loss trajectory matched that of the typically longer DPP-based interventions [36]. More importantly, in the phase 2 follow-up, most of the intervention participants continued to maintain or lose weight.

### Primary Outcome

Full participant recruitment was achieved within 1 year for this difficult-to-reach population. Acceptance criteria presented an interesting conundrum, in that the inclusion and exclusion criteria were stringent and at odds with one another. Participants had to be overweight/obese non-insulin–dependent T2D, with controlled hypertension, yet still capable of walking 30 min per day and willing to deal with a time-consuming protocol and inconvenient blood tests and office visits. Potential participants were approached using various indirect and in-person recruitment strategies, resulting in 185 referrals, yielding only 45 qualified and willing to participate. Yet, despite these recruitment obstacles, the study was feasible.

Recruitment was successful, engagement (office visit attendance) was close to 100%, and a 100% retention rate was achieved, possible due to the culturally adapted intervention and use of a community health worker model to successfully recruit and administer the study. Although cultural adaptation strategies were not quantitatively measured for adherence and feasibility, qualitative process evaluations through semistructured interviews were conducted at the 3-month and 6-month visits to assess cultural acceptability and relevance of the intervention for Filipino Americans. As detailed in a previous publication [17], over half (58%; 26/45) stated that “the culturally tailored support (eg, Filipino research staff) enhanced their engagement” in the study. Furthermore, a majority of participants (64%; 29/45) reported that the intervention helped boost their self-confidence in managing their health. Thus, Filipino American participants deemed the culturally adapted PilAm Go4Health intervention acceptable and relevant.

Adherence to using mobile technology was excellent for wearing the Fitbit to track PA and logging foods to monitor calorie intake. However, adherence for self-monitoring weight was markedly lower. This could be due to the negative feedback that can occur with self-weighing, particularly among those with overweight/obesity. There is debate about self-weighing because in some overweight/obese individuals, it appears to generate negative psychological conditions, such as depression, anxiety, and stress [37,38]. Future studies should assess barriers and facilitators for tracking weight to improve intervention strategies promoting weight loss.

Due to the small sample size of our study, we were unable to assess the relative contribution of the mobile app use to weight loss outcomes. Such analyses may be feasible in future studies with a larger sample size. Nevertheless, the PilAm Go4Health adherence data add to the body of knowledge that mobile apps are useful for tracking health behaviors in weight loss interventions.

More importantly, our participants’ mean age was 57.6 years, which demonstrated that older adults can successfully learn and use mobile technology to self-monitor health. In our previous publication, overall, participants highly endorsed and adopted the Fitbit as a means for tracking PA and reported that the mobile technology helped improve accountability for monitoring target health behaviors [17]. Previous studies have shown that a majority of older adults go online and own a smartphone, but few engage in using mobile technology [39,40]. Future mHealth lifestyle intervention studies should evaluate whether older adults will continue to engage in the use of mobile technology after receiving an mHealth-based intervention.

### Secondary Outcomes

Evidence indicates that weight loss of 5% to 7% by 6 months is associated with preventing or reducing T2D and cardiovascular risks [10,40-42]. Even a modest weight loss of 5% in patients with T2D is associated with significant clinical improvements (eg, systolic blood pressure, glucose, HbA1c, and triglycerides). Due to the short 3-month intervention duration, PilAm Go4Health study participants may not have had sufficient time to achieve the 5% weight loss goal set forth in other 6-month weight loss lifestyle interventions [36].

Overall, 24% (11/45) of intervention group and waitlist group participants achieved the study’s primary 5% weight loss goal after completing PilAm Go4Health, and 31% (14/45) achieved a 2% to 5 % weight loss (Table 6). However, considering the reduced number of office visits and educational meetings condensed into 3 months, the trajectory for PilAm Go4Health participant weight loss rates was similar to those of longer DPP-based studies [36]. Increasing the PilAm Go4Health duration to 6 months may be necessary to achieve the 5% to 7% weight reduction for optimum health benefits.

Notably, compared with phase 1 when only 18% (4/22) of intervention participants achieved the 5% weight loss goal, nearly twice the number of waitlist participants, 30% (7/23), achieved their 5% weight loss goal in phase 2. When waitlist participants received PilAm Go4Health, they had already been self-monitoring PA steps for the prior 3 months. During phase 1, the waitlist group was asked to only self-monitor PA steps using the Fitbit app. This prior PA tracking behavior may have contributed to the greater number of waitlist participants achieving the 5% weight loss goal in phase 2 compared with the intervention group.

### Other Outcomes

Our study results highlight the important relationship between weight management and diabetes control. Not surprising, the other outcomes of weight (kg), waist circumference, and BMI mirrored the results of the secondary outcome for reduced percent weight over 3 months when both study arms received the PilAm Go4Health. Similarly, the PilAm Go4Health had a large effect on PA measured by increased steps over time. Furthermore, improvements in fasting glucose and HbA1c give promise to the efficacy of the PilAm Go4Health mHealth intervention to enhance diabetes self-management. There were clear improvements in diabetes self-management and control as reflected in the significant cross-level interactions (with the exception of phase 1 for HbA1c); however, long-term studies are needed to detect whether serum levels for diabetes control can be improved and sustained.

### Strengths

PilAm Go4Health intervention program has several noteworthy strengths. This is one of the first rigorous lifestyle intervention studies focused on Filipinos with obesity and T2D to reduce further cardiovascular-metabolic complications. The efficacy of the PilAm Go4Health program was evident in the (1) 100% (45/45) participant study completion rate, demonstrating the excellent participant recruitment and engagement of this hard-to-reach Filipino population; (2) ability to promote weight loss among Filipino Americans with overweight and T2D (in both study arms receiving the intervention) in only 3 months; and (3) sustained intervention group weight loss in the subsequent 3 months.

There are multiple intervention factors (eg, intensity, Facebook, self-monitoring behaviors, cultural adaptation) that may have contributed to the PilAm Go4Health potential efficacy. Although relative contributions of each factor are unknown, previous studies indicate that each may have had a positive impact on the outcomes. First, intensive interventions with multiple components have shown that participants are able to lose 5% of their baseline weight over short durations [36,43,44]. PilAm Go4Health findings are consistent with these studies.

Second, evidence indicates that self-monitoring lifestyle behaviors improve weight loss and health outcomes [7,45]. Furthermore, higher adherence to activity tracking was associated with greater weight loss and increased PA [46,47]. Our findings support these 2 premises in that the waitlist group’s self-monitoring (tracking) of PA alone in phase 1 was not sufficient to achieve a significant weight reduction by 3 months. However, in phase 2, when the waitlist group received the PilAm Go4Health intervention, tracking PA in combination with tracking food calories and weight resulted in significant weight reductions or weight stabilization in 3 months.

A third intervention factor was cultural relevance, an integral element of the PilAm Go4Health design. Culturally relevant interventions have been shown to improve health outcomes, especially in diverse immigrant populations [48,49]. Cultural tailoring is an important strategy to improve recruitment, engagement, and retention in a hard-to-reach vulnerable population [18,22]. Cultural adaptation strategies used in the study design reflected Filipino family and community preferences by welcoming family members at research office visits for in-person social support and incorporating a private Facebook group for virtual social support with peers. Therefore, these culturally adaptations may have influenced the weight loss achieved by a primarily immigrant population.

Incorporating mobile technology was a fourth factor in the PilAm Go4Health study design. Including a mobile phone app to supplement standard lifestyle counseling had positive impact on PA and diet, even over short 3-month intervention periods such as that of the PilAm Go4Health [43,50]. Furthermore, our findings are consistent with other studies that show technology-based interventions are feasible, acceptable, and efficacious among older adults [16,51]. On the basis of FA’s prolific use of mobile technology and social media [13], PilAm Go4Health incorporated this technology to promote participant engagement and motivate self-monitoring of lifestyle behaviors to achieve weight loss goals. The addition of virtual social networking via Facebook in our study may have contributed to adherence for tracking health behaviors. Virtual social media has also been shown to improve health outcomes. For example, Facebook has also been used in weight loss interventions, with positive results among overweight/obese adults and college students [52,53].

Our study supports evidence that older individuals can successfully use mobile technology to improve diabetes self-management. The Pew Research Center reported that older adults with higher education more easily adopt mobile technology compared with less-educated older adults [51]. On the basis of our study’s previously reported qualitative outcome [17], our highly educated older Filipino American adults seemed to readily adopt the mobile technology to track health behaviors for diabetes management. The mobile technology used in our study may have influenced adherence to healthy behaviors, contributing to weight reduction and improvements in other health outcomes. Further research is needed to evaluate the relative influence of the mHealth components used in the PilAm Go4Health program.

### Limitations

There are several limitations of note. The duration of this pilot RCT 3-month lifestyle intervention with a 3-month follow-up was shorter than a typical DPP-based 6-month weight loss program. This may have influenced participants’ ability to achieve the overall study’s 5% weight loss goal. The small sample size consisted of a highly educated immigrant Filipino population from a geographic area of Northern California, limiting the internal validity and generalizability. The study was also limited to those who were English literate and owned smartphones with Internet access. This may have resulted in a biased sample excluding non-English speakers and those less likely to afford mobile devices requiring Internet service. The sample size also limited statistical analyses of this multifactorial intervention program, restricting the ability to determine the relative contribution of each factor influencing weight loss and other outcomes. Nevertheless, many secondary/other outcomes (eg, percent weight, weight [kg], and fasting glucose change) were statistically significant, indicating that power (and therefore the sample size) was sufficient to support the potential efficacy of the PilAm Go4Health [54,55].

The run-in period to assess participant eligibility may have biased retention levels and study outcomes as it may have excluded noncompliant potential participants. However, out of 52 potential PilAm Go4Health participants completing the run-in, only 4% (2/52) were categorized as noncompliant (see Figure 1). Furthermore, a recent meta-analysis of interventions in which weight loss was the primary outcome showed studies did not differ significantly in weight loss with or without a run-in period [56] and thus did not compromise generalizability.

### Implications

These study findings have practical clinical implications for health care providers. As the obesity epidemic grows, health care providers should routinely address the issues of obesity and inactivity that are associated with poor health outcomes. Our results will help inform clinicians about commercially available mHealth tools and social media for patients’ use to improve health outcomes. Clinicians can tailor patient weight loss goals using these tools to promote engagement and adherence to healthy lifestyle behaviors. In our study, real-time feedback from the Fitbit accelerometers along with the associated mHealth app with diary for tracking weight and food/calories may have been an important motivational factor. Utilizing Facebook capabilities for virtual social support among peers in tandem with health education postings may have also influenced improvements in health behavior change [34].

### Conclusions

PilAm Go4Health demonstrated that a mobile technology–based culturally adapted lifestyle intervention was feasible and potentially efficacious in weight reduction among older understudied Filipino Americans with obesity and T2D. Results are promising for targeted, culturally tailored lifestyle interventions in achieving short-term weight loss. Therefore, a larger RCT is warranted to test effectiveness of the PilAm Go4Health in maintaining long-term weight loss to reduce T2D and cardiovascular-metabolic risks in this vulnerable population.

## Appendix

Multimedia Appendix 1 CONSORT‐EHEALTH checklist (V 1.6.1).

## Abbreviations

BMI: body mass index
BC CI: bias-corrected bootstrapped confidence interval
CDC: Centers for Disease Control and Prevention
DPP: Diabetes Prevention Program
mHealth: mobile health
PA: physical activity
PilAm Go4Health: Pilipino Americans Go4Health
RCT: randomized controlled trial
T2D: type 2 diabetes
