# Correction: A Feasible and Efficacious Mobile-Phone Based Lifestyle Intervention for Filipino Americans with Type 2 Diabetes: Randomized Controlled Trial

**DOI:** 10.2196/12784

**Published:** 2018-12-21

**Authors:** Melinda S Bender, Bruce A Cooper, Linda G Park, Sara Padash, Shoshana Arai

**Affiliations:** 1 Family Health Care Nursing Department School of Nursing University of California San Francisco San Francisco, CA United States; 2 Office of the Dean and Administration School of Nursing University of California San Francisco San Francisco, CA United States; 3 Community Health Services School of Nursing University of California San Francisco San Francisco, CA United States; 4 School of Nursing University of San Francisco San Francisco, CA United States

The authors of the paper “A Feasible and Efficacious Mobile-Phone Based Lifestyle Intervention for Filipino Americans with Type 2 Diabetes: Randomized Controlled Trial” (JMIR Diabetes 2017;2(2)e30) made a mistake in the final stage of proofreading. The metadata incorrectly designated Linda G Park as a PNP instead of a PhD. Her correct designation is PhD, RN, FNP-BC, FAHA.

The correction will appear in the online version of the paper on the JMIR website on December 21, 2018, together with the publication of this correction notice. Because this was made after submission to PubMed, PubMed Central, and other full-text repositories, the corrected article also has been resubmitted to those repositories.

